# Time to diagnosis and associated costs of an outpatient vs inpatient setting in the diagnosis of lymphoma: a retrospective study of a large cohort of major lymphoma subtypes in Spain

**DOI:** 10.1186/s12885-018-4187-y

**Published:** 2018-03-12

**Authors:** Xavier Bosch, Carmen Sanclemente-Ansó, Ona Escoda, Esther Monclús, Jonathan Franco-Vanegas, Pedro Moreno, Mar Guerra-García, Neus Guasch, Alfons López-Soto

**Affiliations:** 1 Quick Diagnosis Unit, Adult Day Care Center, Hospital Clínic, University of Barcelona, Villarroel 170, 08036 Barcelona, Spain; 2 Quick Diagnosis Unit, Department of Internal Medicine, Hospital of Bellvitge, University of Barcelona, Barcelona, Spain; 3 Department of Internal Medicine, Hospital Clínic, University of Barcelona, Barcelona, Spain; 4 Adult Day Care Center, Hospital Clínic, University of Barcelona, Villarroel 170, 08036 Barcelona, Spain; 5 Quick Diagnosis Unit, Department of Internal Medicine, Hospital Clínic, University of Barcelona, Villarroel 170, 08036 Barcelona, Spain

**Keywords:** Lymphoma, Outpatient, Inpatient, Time to diagnosis, Diagnosis, Length of stay, Excisional biopsy, Fine-needle aspiration cytology, Emergency departments, Primary care

## Abstract

**Background:**

Mainly because of the diversity of clinical presentations, diagnostic delays in lymphoma can be excessive. The time spent in primary care before referral to the specialist may be relatively short compared with the interval between hospital appointment and diagnosis. Although studies have examined the diagnostic intervals and referral patterns of patients with lymphoma, the time to diagnosis of outpatient compared to inpatient settings and the costs incurred are unknown.

**Methods:**

We performed a retrospective study at two academic hospitals to evaluate the time to diagnosis and associated costs of hospital-based outpatient diagnostic clinics or conventional hospitalization in four representative lymphoma subtypes. The frequency, clinical and prognostic features of each lymphoma subtype and the activities of the two settings were analyzed. The costs incurred during the evaluation were compared by microcosting analysis.

**Results:**

A total of 1779 patients diagnosed between 2006 and 2016 with classical Hodgkin, large B-cell, follicular, and mature nodal peripheral T-cell lymphomas were identified. Clinically aggressive subtypes including large B-cell and peripheral T-cell lymphomas were more commonly diagnosed in inpatients than in outpatients (39.1 vs 31.2% and 18.9 vs 13.5%, respectively). For each lymphoma subtype, inpatients were older and more likely than outpatients to have systemic symptoms, worse performance status, more advanced Ann Arbor stages, and high-risk prognostic scores. The admission time for diagnosis (i.e. from admission to excisional biopsy) of inpatients was significantly shorter than the time to diagnosis of outpatients (12.3 [3.3] vs 16.2 [2.7] days; *P* < .001). Microcosting revealed a mean cost of €4039.56 (513.02) per inpatient and of €1408.48 (197.32) per outpatient, or a difference of €2631.08 per patient.

**Conclusions:**

Although diagnosis of lymphoma was quicker with hospitalization, the outpatient approach seems to be cost-effective and not detrimental. Despite the considerable savings with the latter approach, there may be hospitalization-associated factors which may not be properly managed in an outpatient unit (e.g. aggressive lymphomas with severe symptoms) and the cost analysis did not account for this potentially added value. While outcomes were not analyzed in this study, the impact on patient outcome of an outpatient vs inpatient diagnostic setting may represent a challenging future research.

## Background

In the absence of pathological lumps, lymphomas may pose a difficult diagnostic challenge. Patients presenting with enlarged lymphadenopathy are straightforwardly diagnosed. However, others present with systemic symptoms such as long-lasting fever and progressive weight loss and a worsening performance status. And in the middle of the spectrum are patients who complain of nonspecific or subtle symptoms [1].

Studies investigating the mean duration of intervals on the illness paths of patients with lymphomas have shown that whereas the patient interval (i.e. from onset of symptoms to first medical contact) constitutes the longest individual interval, the primary care (PC) interval (i.e. time spent in PC before referral to the specialist) is highly variable depending not only on the PC physician experience but also on the broad range of presenting symptoms [1, 2]. Because symptoms are often seen in PC in association with other more common and less serious conditions, patients are referred to a wide range of specialists, with a few referred directly to the appropriate secondary specialist [3]. A case series study conducted in patients aged > 25 years with newly diagnosed lymphomas who were first seen in PC revealed that only 12% were directly referred to the hematologist and that this specialist reached a quicker diagnosis than any other specialist [3]. Indeed, diagnostic delays can be excessive, and studies have shown that the PC interval may be relatively short compared with the diagnostic interval (i.e. from first specialist appointment to actual diagnosis) [2]. The heterogeneity of presentations and symptoms severity reflects the heterogeneous assortment of lymphoma subtypes and their aggressive or more indolent behavior [1, 2, 4, 5].

In the 2000 recommendations of the United Kingdom (UK) to improve cancer survival, hematological malignancies were considered as a single entity [6]. When considering referral for specialist assessment, the updated 2015 referral guidelines of the National Institute for Health and Care Excellence (NICE) highlighted unexplained lymphadenopathy and/or splenomegaly as well as other symptoms and signs that may suggest Hodgkin and non-Hodgkin lymphoma and that deserve further investigation [7]. The updated guidelines included separate recommendations for adults and for children and young people (aged 16-24 years) to indicate that there are different referral pathways. However, the significance and ranking of each particular sign and symptom is not disclosed [7]. Although quick access lymph node diagnostic clinics recommended by NICE in 2003 to reduce the time to diagnosis in patients with suspected lymphoma [8] appeared a more useful, simplified referral pathway [9], the challenge of appropriate referral remains in those patients who do not present with lymphadenopathy.

Many of the above findings come from the UK. However, they may well be generalizable to other health care systems where the patient first sees a PC physician [10] such as the Spanish public health care system.

To reduce diagnostic delays of patients with potentially serious diseases, the Spanish health care system created in the mid-2000s the so-called quick diagnosis units (QDUs). These hospital based-outpatient facilities have proven to be cost-effective alternatives to conventional hospitalization for diagnostic workup of disorders such as highly suspected cancer, severe anemia, or fever of unknown origin [11–13].

Largely because of long-delayed investigations ordered by PC physicians, these patients were traditionally hospitalized to speed-up the diagnostic process. However, most of them were too well to justify admission as just waited for examinations without receiving actual therapy [14–16]. While many QDUs were implemented during the recent economic regression to reduce the huge expenses associated with hospitalization [17–19], admission for workup is one of the most common reasons for inappropriate hospitalizations in Spain and elsewhere [19–23].

In contrast to the more focused approach of specialists at units such as the UK one-stop diagnostic clinics [9, 24], the versatility of general internists for the diagnosis of conditions characterized by nonspecific symptoms such as unintentional weight loss, fatigue or unexplained fever explains why QDUs are predominantly run by these physicians [11–13, 25].

Although reported data on these units are limited, several advantages over hospitalization have been established: in addition to ensuring a time to diagnosis similar to the length of stay for the same evaluable conditions, they are known to decrease emergency department (ED) referrals from PC easing ED overcrowding, are associated with higher patient satisfaction scores, and are considerably cost-saving [11–13, 15, 16, 25–29]. Yet to be evaluated, patients’ physical performance should allow them to travel from home to hospital and back for visits and investigations.

Because an acceptable performance status argues in general against admission of subjects who are eventually diagnosed with lymphoma after referral to QDUs for investigation of typical or nontypical manifestations, these outpatient units appear a proper setting for their assessment. However, no study has examined the time to diagnosis and associated costs of an outpatient compared to an inpatient setting. The main purpose of this retrospective study was to investigate the time to diagnosis of a hospital-based outpatient or inpatient setting in four major subtypes of lymphomas and the costs incurred by both clinical settings in the diagnostic process. A further goal was to investigate the frequency, clinical, and prognostic features of each lymphoma subtype according to an outpatient or inpatient diagnosis.

## Methods

### Settings and study population

The QDU of the Hospital Clínic (QDU (1)), a public 855-bed tertiary university hospital in Barcelona (Spain) with a reference population of almost 550,000, the inpatient wards of the internal medicine department of this hospital, and the QDU of the Hospital of Bellvitge (QDU (2)), a public 750-bed tertiary university hospital near Barcelona with a reference population of almost 350,000, participated in the study. All patients had been referred to the three settings from the respective PCs and EDs between January 2006 and September 2016. The referral criteria of the two outpatient units are essentially the same [14, 15, 30]. For the purpose of this study, both outpatient units were combined, and the analyzed patients were considered a single outpatient cohort (herein referred to as ‘outpatients’). The study was approved by the Comitè d’ Ètica de la Investigació Clínica (Clinical Research Ethics Committee) of the Hospital Clínic and the Comitè d’ Ètica i Assajos Clínics (Ethics and Clinical Assays Committee) of the University Hospital of Bellvitge. The ethics committees waived the need for written informed consent because no intervention was involved and the retrospective analysis of clinical data.

To analyze a homogeneous and representative sample of major lymphoma subtypes, cases included were patients aged ≥ 18 years with classical Hodgkin lymphoma, large B-cell lymphoma, follicular lymphoma, and mature nodal peripheral T-cell lymphoma (comprising peripheral T-cell lymphoma, not otherwise specified [NOS], angioimmunoblastic T-cell lymphoma, and anaplastic large cell lymphoma, anaplastic lymphoma kinase [ALK]-negative). All cases were diagnosed according to the 2008 World Health Organization classification of lymphoid neoplasms [31] and codified according to the International Classification of Diseases for Oncology (3rd edition; ICD-O-3) [32].

Pathologists at the Hospital Clínic and Hospital of Bellvitge selected and reviewed histopathological data of consecutive cases with these diagnoses, and attending and resident physicians from the outpatient and inpatient units reviewed the medical records of all patients and entered the following data into an electronic database: 1) individual frequency of lymphoma subtypes; 2) general demographic and clinical information including clinical manifestations at presentation (lymphadenopathy, systemic, pain and chest symptoms, or other symptoms or signs), relevant laboratory data (white blood cell count [including absolute lymphocyte count], erythrocyte count, platelet count, hemoglobin level, serum liver enzymes and lactate dehydrogenase [LDH], serum albumin, erythrocyte sedimentation rate, and C-reactive protein), and imaging reports; and 3) specific lymphoma information including Ann Arbor stage, Eastern Cooperative Oncology Group (ECOG) performance score, B symptoms, extranodal disease, bulky disease (maximum tumor dimension ≥ 10 cm) for classical Hodgkin and large B-cell lymphomas, international prognostic score (IPS) for advanced-stage classical Hodgkin lymphoma [33], international prognostic index (IPI) score for large B-cell and nodal peripheral T-cell lymphomas [34], follicular lymphoma international prognostic index (FLIPI) score for follicular lymphoma [35], and histologic grading of follicular lymphoma. The medical records of patients without explicit registered information on ECOG performance, bulky disease, and IPS, IPI, and FLIPI scores were carefully assessed to determine these parameters when all individual required data were available. Otherwise, the actual number and percentage of cases relative to the total number of cases with complete data for each parameter was entered into the database.

Patients without a biopsy-proven diagnosis and those with human immunodeficiency virus infection, a prior diagnosis of another lymphoma, incomplete clinical or pathological information, lost to follow-up, or dead before initial staging were excluded.

### Fine-needle aspiration cytology

Although all study patients underwent an excisional biopsy, we calculated in a separate analysis the number of fine-needle aspiration cytologies (FNACs) performed and the proportion of suspected or compatible diagnosis of lymphoma according to cytomorphology and/or flow cytometry studies. In particular, following a systematic approach [36], a fast FNAC was commonly performed in patients (principally outpatients) with easily accessible lumps, then followed by a mandatory excisional biopsy in those with a suspicious/compatible lymphoma diagnosis. While both FNAC and biopsy were ordered by physicians from the inpatient and outpatient settings, to save time, outpatients with a positive FNAC result were immediately referred to the hematology (for QDU (1)) and the oncology (for QDU (2)) outpatient clinics, without need to wait for the biopsy being performed at time of referral. In contrast, irrespective of FNAC, all inpatients were discharged once the biopsy had been performed and its results were available. For consistency, cytologists at the two hospitals reviewed cytologic data of patients with a positive FNAC result and compared the cytologic and histopathological findings on an individual basis.

### Activities of the inpatient and outpatient settings

The activities of the inpatient and outpatient units during the diagnostic evaluation were reviewed and registered. These included intervals between referral and first outpatient appointments/inpatient admissions, intervals between first outpatient appointments/inpatient admissions and dates of FNACs and excisional biopsies, successive/first visit ratio and time to diagnosis in outpatients, length of stay in inpatients, and onward referrals upon discharge of outpatients and inpatients. Additionally, although disease staging was systematically performed by the hematologist or the oncologist, physicians from the inpatient and outpatient units ordered a positron emission tomography-computed tomography (PET-CT) scan when lymphoma was suspected or confirmed.

### QDU time for diagnosis and admission time for diagnosis

As mentioned, outpatients with a positive FNAC diagnosis were discharged from the outpatient environment without an excisional biopsy still being done. To allow for a better meaning and equivalent measure of time to diagnosis between outpatients and inpatients, we defined QDU time for diagnosis as the time elapsed between the first outpatient visit and the excisional/diagnostic biopsy, and admission time for diagnosis (instead of length of stay) as the actual time elapsed between inpatient admission and the excisional biopsy. This was made to try to deal with the differences in patient- and lymphoma-related characteristics that may influence comparison between results. More specifically, because the duration of hospitalization is likely impacted by inpatient factors (i.e. inpatients may be older, sicker, and have presented with more symptoms and advanced stages of disease than outpatients), length of stay may not be a precise reflection of the time needed to evaluate and diagnose a patient with lymphoma. Indeed, inpatients may require a longer hospitalization both to expedite lymphoma diagnosis and for symptom management and, as such, they are more prone than outpatients to incur greater care-related costs.

### Resource use data collection and cost analysis

Costs of outpatients and inpatients were analyzed and compared with the microcosting method, often considered as a paradigm of hospital service costs, since all relevant cost components are precisely determined [37–39]. The microcosting methodology used by us for other disorders has been described elsewhere [11–13, 27]. In brief, resource use for each patient evaluated was obtained. Resource use data collected included all laboratory tests, cytologies, biopsies, imaging studies, and any other diagnostic procedure performed. The excisional biopsy requested for outpatients who were discharged with an onward referral if the FNAC result was positive was also included in the cost analysis. Data were also gathered on pharmaceuticals and consumables, therapeutic procedures, adverse events, and consultations. Only treatments other than lymphoma-specific treatments (i.e. treatment of patient’s symptoms) were included in the analysis. Thus, lymphoma-specific therapies such as chemotherapy and biologic agents were started by the hematologist or the oncologist after their first patient assessment once inpatients and outpatients had been discharged and referred to them. Costs of all individual resource items of outpatients and inpatients were obtained from the institutional information systems of the Hospital Clínic and Hospital of Bellvitge (bottom-up microcosting). For outpatients, the cost of an average outpatient consultation was determined according to officially established Catalan Health Service fees. In addition, the cost of each type of diagnostic investigation corresponded to hospital tariffs for QDU (1) outpatients and Catalan Health Service fees for QDU (2) outpatients. For inpatients, the costs of diagnostic tests, which were based on the same costs used for QDU (1) outpatients, were also computed.

The microcosting analysis also incorporated fractions of all staff wages. Particularly, QDU (1) is integrated in the internal medicine department of the Hospital Clínic and its staff includes a full-time consultant internist, a senior internal medicine resident, a full-time nurse, a part-time nurse coordinator, and two part-time secretaries. The unit is open 5 h a day, 4 days a week. Furthermore, QDU (2) is also integrated in the internal medicine department of the Hospital of Bellvitge and its staff includes a part-time consultant internist and a part-time nurse. This unit is open 7 h a day, 2 days a week. Finally, staff in the inpatient setting (three wards) includes two full-time consultant internists, three residents, a full-time nurse coordinator, three teams of three full-time nurses and three teams of two full-time nursing assistants (8-h daily shifts), and a full-time secretary.

With an outpatient approach, patients (and any caregiver) may incur in more costs. Thus, indirect costs and costs borne by patients were not included in the cost analysis. Depreciation of fixed costs was included in the final analysis.

Mean number of visits, cost per visit and cost per patient from QDU (1) and (2), and mean admission time for diagnosis, cost per day of stay and cost per patient from the inpatient wards were computed and compared. Costs of referring agencies were excluded. All costs were adjusted for the year of collection (2006-2016) to reflect 2016 Euros (€). Final costs and cost differences are presented in 2016 Euros.

### Statistical analysis

Categorical data were compared using the chi-square test or the Fisher’s exact test, as appropriate, and are expressed as absolute frequencies (%). Continuous variables with a normal distribution were compared using the *t*-test, and are expressed as means with standard deviations (SD). The nonparametric Mann-Whitney *U* test was used, when appropriate, to compare continuous variables with skewed distributions. The extent and nature of any missing data was also included in the analysis. Statistical significance was set at *P* < .05. Analyses were performed using the SPSS software (version 21.0) (SPSS, Chicago, USA).

## Results

### General characteristics of study population

**Fig. 1:**
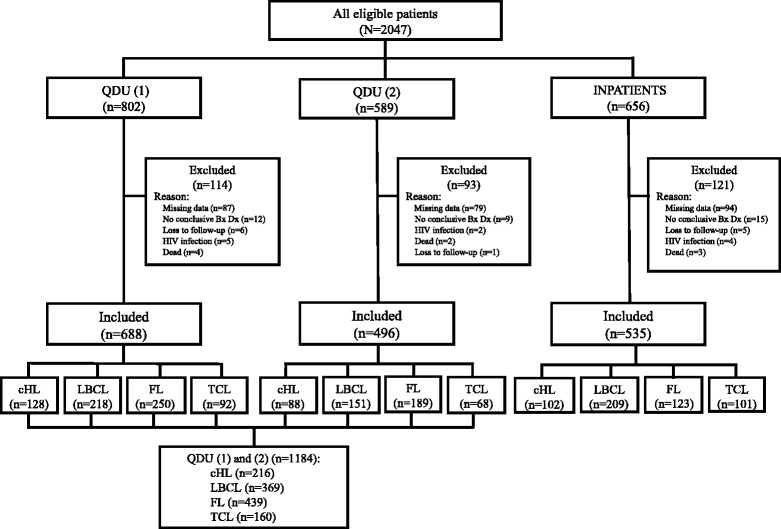
Flowchart of patients included in the study. *Abbreviations*: *QDU (1)* cohort of patients from the quick diagnosis unit of the Hospital Clínic, *QDU (2)* cohort of patients from the quick diagnosis unit of the Hospital of Bellvitge, *QDU (1) and (2)* combined cohort of patients from the quick diagnosis units of the Hospital Clínic and the Hospital of Bellvitge, *Bx* biopsy, *Dx* diagnosis, *HIV* human immunodeficiency virus, *cHL* classical Hodgkin lymphoma, *LBCL* large B-cell lymphoma, *FL* follicular lymphoma, *TCL* mature nodal peripheral T-cell lymphoma

Of 2047 eligible patients, 328 were excluded. Figure 1 shows the number of initially eligible patients from QDU (1), QDU (2), and inpatient wards and the causes for their exclusion. The main reasons for exclusion were incomplete clinical or pathological information and absence of a conclusive pathological diagnosis. After exclusion, 1719 patients comprising 1184 outpatients (688 from QDU (1) and 496 from QDU (2)) and 535 inpatients were available for the analysis (Fig. 1). The general characteristics of the whole population is shown in Table 1. Mean age was 63.4 (17.4) years and 55.4% were males. There were minor differences in patient characteristics between QDU (1) and QDU (2) outpatient cohorts (Tables 1 and 2). Inpatients were older and more likely to be males than outpatients. Nearly 65% of patients presented with lymphadenopathy, which was more frequent in outpatients than in inpatients. Just above 25% of patients had systemic symptoms and these were more common in inpatients (35.7%) than in outpatients (21.8%). The main referral source of inpatients was ED (i.e. emergency admissions) (68.4%) and it was PCs in outpatients (75.5%). Expectedly, inpatients waited less than 24 h to be admitted, whereas time to first visit in outpatients was significantly longer (0.6 [0.3] vs 1.7 [1.1) days; *P* < .001) (Table 1). The admission time for diagnosis of inpatients was significantly shorter than the QDU time for diagnosis of outpatients (12.3 [3.3] vs 16.2 [2.7] days; *P* < .001). An FNAC was performed in 935 (54.4%) patients (766 [64.7%] outpatients and 169 [31.6%] inpatients) yielding an overall suspicious/compatible lymphoma diagnosis in 65.3% with no false positive result in any patient after considering the biopsy findings. The rate of positive FNAC results was slightly, albeit nonsignificantly, higher in outpatients than in inpatients. Specifically, 65.7% of outpatients vs 63.9% of inpatients had a suspected/compatible lymphoma by FNAC (*P* = .102). The mean time to biopsy was substantially longer in outpatients (7.4 [1.8] days) than in inpatients (3.5 [1.1] days) (*P* < .001). After diagnosis, most patients were referred to outpatient specialist clinics and inpatients more often received direct palliative care after discharge than outpatients. There was ≤ 3% of missing data on the variables waiting time to first QDU visit, waiting time to admission, time to FNAC, and time to excisional biopsy (see footnote of Table 1).

**Table 1:** General characteristics of study patients^a^

Characteristic	Total (*N* = 1719)	QDU (1) OPs (*n* = 688)	QDU (2) OPs (*n* = 496)	Total OPs *1* (*n* = 1184)	IPs *2* (*n* = 535)	*P* value *1* vs *2*
Age (years), mean (SD)	63.4 (17.4)	62.7 (13.8)	61.8 (11.5)	62.3 (14.7)	65.6 (12.2)	<.001
Sex, *n* (%)						
Females	768 (44.6)	318 (46.2)	227 (45.8)	545 (46.0)	223 (41.7)	<.001
Males	951 (55.4)	370 (53.8)	269 (54.2)	639 (54.0)	312 (58.3)	<.001
Clinical manifestations, *n* (%)^b^						
Lymphadenopathy	1112 (64.7)	487 (70.8)	358 (72.2)	845 (71.4)	267 (49.9)	<.001
Systemic symptoms^c^	449 (26.1)	155 (22.5)	103 (20.8)	258 (21.8)	191 (35.7)	<.001
Pain symptoms^d^	89 (5.2)	26 (3.8)	17 (3.4)	43 (3.6)	46 (8.6)	<.001
Chest symptoms^e^	45 (2.6)	14 (2.0)	12 (2.4)	26 (2.2)	19 (3.6)	.128
Other symptoms/signs^f^	24 (1.4)	6 (0.9)	6 (1.2)	12 (1.0)	12 (2.2)	.139
Referral sources, *n* (%)						
Emergency department	656 (38.2)	182 (26.5)	108 (21.8)	290 (24.5)	366 (68.4)	<.001
Primary care	1063 (61.8)	506 (73.5)	388 (78.2)	894 (75.5)	169 (31.6)	<.001
Waiting time to first QDU visit/admission (days), mean (SD)^g^		1.7 (0.8)	1.8 (0.7)	1.7 (1.1)	0.6 (0.3)	<.001
Successive/first visit ratio		2.29	2.12	2.22		
QDU time for diagnosis/admission time for diagnosis (days), mean (SD)^h^		16.8 (2.5)	15.4 (2.2)	16.2 (2.7)	12.3 (3.3)	<.001
Suspected/compatible lymphoma by FNAC, *n* (%)/total n^i^	611 (65.3)/935	302 (67.3)/449	201 (63.4)/317	503 (65.7)/766	108 (63.9)/169	.102
Time to FNAC (days), mean (SD)^j^	1.2 (0.9)	1.5 (0.8)	1.0 (0.5)	1.3 (0.9)	1.2 (0.6)	.183
Time to excisional biopsy (days), mean (SD)^k^	5.7 (1.7)	7.5 (1.6)	7.1 (1.4)	7.4 (1.8)	3.5 (1.1)	<.001
Onward referrals, *n* (%)						
Outpatient specialist clinics	1622 (94.4)	654 (95.1)	476 (96.0)	1130 (95.4)	492 (92.0)	.046
Primary care	47 (2.7)	21 (3.1)	15 (3.0)	36 (3.0)	11 (2.1)	.169
Palliative care	50 (2.9)	13 (1.9)	5 (1.0)	18 (1.5)	32 (6.0)	<.001

*Abbreviations*: *QDU (1) OPs* cohort of outpatients from the quick diagnosis unit of the Hospital Clínic; *QDU (2) OPs* cohort of outpatients from the quick diagnosis unit of the Hospital of Bellvitge; *Total OPs* total number of outpatients from the quick diagnosis units of the Hospital Clínic and the Hospital of Bellvitge*; IPs* cohort of hospitalized patients of the Hospital Clínic; *FNAC* fine-needle aspiration cytology

^a^Lymphoma subtypes are not included; ^b^Single or combined; ^c^Mainly include intense tiredness, abnormal sweating at night, unintentional weight loss, nausea, and anorexia; ^d^Mainly abdominal pain; ^e^Mainly include shortness of breath, cough, and sore throat; ^f^Include, among others, pruritus, bowel symptoms, unusually thirsty, fever, and incidental findings on imaging studies; ^g^Intervals between referral and first QDU appointment and hospital admission, respectively; ^h^Intervals between first QDU visit/hospital admission and excisional biopsies; ^i^n (%)/total n: actual number and percentage of cases relative to the total number of available cases; ^j^Interval between ordering FNAC and the procedure being done; ^k^Interval between ordering biopsy and the procedure being done Missing data: variables ‘waiting time to first QDU visit’ (total OPs = 19), ‘waiting time to admission’ (IPs = 10), ‘time to FNAC’ (total OPs = 24, IPs = 16), ‘time to excisional biopsy’ (total OPs = 6, IPs = 4)

**Table 2:** Frequency of lymphoma subtypes in outpatient and inpatient cohorts

Subtype^a^	Total (*N* = 1719)	QDU (1) OPs (*n* = 688)	QDU (2) OPs (*n* = 496)	Total OPs *1* (*n* = 1184)	IPs *2* (*n* = 535)	*P* value *1* vs *2*
Classical Hodgkin lymphoma	318 (18.5)	128 (18.6)	88 (17.7)	216 (18.2)	102 (19.1)	.164
Nodular sclerosis, NOS^b^	235 (73.9)	96 (75.0)	66 (75.0)	162 (75.0)	73 (71.6)	.045
Mixed cellularity, NOS^c^	37 (11.6)	16 (12.5)	10 (11.4)	26 (12.0)	11 (10.8)	.135
Lymphocyte rich^d^	13 (4.1)	5 (3.9)	3 (3.4)	8 (3.7)	5 (4.9)	.140
Lymphocyte depletion, NOS^e^	4 (1.3)	1 (0.8)	0 (0.0)	1 (0.5)	3 (2.9)	.078
Classical Hodgkin lymphoma, NOS^f^	29 (9.1)	10 (7.8)	9 (10.2)	19 (8.8)	10 (9.8)	.156
Large B-cell lymphoma^g^	578 (33.6)	218 (31.7)	151 (30.4)	369 (31.2)	209 (39.1)	<.001
Follicular lymphoma, NOS^h^	562 (32.7)	250 (36.3)	189 (38.1)	439 (37.1)	123 (23.0)	<.001
Mature nodal peripheral T-cell lymphoma^i^	261 (15.2)	92 (13.4)	68 (13.7)	160 (13.5)	101 (18.9)	<.001
Peripheral T-cell lymphoma, NOS^j^	147 (56.3)	50 (54.3)	37 (54.4)	87 (54.4)	60 (59.4)	<.001
Angioimmunoblastic T-cell lymphoma^k^	77 (29.5)	28 (30.4)	21 (30.9)	49 (30.6)	28 (27.7)	.076
Anaplastic large cell lymphoma, ALK-negative^l^	37 (14.2)	14 (15.2)	10 (14.7)	24 (15.0)	13 (12.9)	.086

*Abbreviations*: *NOS* not otherwise specified; *ALK* anaplastic lymphoma kinase. For other abbreviations, see Table 1

^a^The ICD-O-3 codes of the International Classification of Diseases for Oncology, 3rd edition [32], are shown for each lymphoma subtype; ^b^9663/3; ^c^9652/3; ^d^9651/3; ^e^9653/3; ^f^9650/3; ^g^9680/3, 9684/3, 9735/3, 9688/3, and 9679/3 (anaplastic large B-cell lymphoma, diffuse large B-cell lymphoma associated with chronic inflammation, primary diffuse large B-cell lymphoma of the CNS, primary cutaneous DLBCL leg type, and EBV positive diffuse large B-cell lymphoma of the elderly were excluded); ^h^9690/3; ^i^9702/3; ^j^9702/3; ^k^9705/3; ^l^9702/3

### Frequency of lymphoma subtypes in outpatient and inpatient settings

Table 2 shows the total number of lymphoma subtypes and their frequency in each cohort of patients. Overall, large B-cell lymphoma was the leading subtype of lymphoma (33.6%) and follicular lymphoma ensued (32.7%). Large B-cell lymphoma was more frequent in inpatients than in outpatients (39.1% vs 31.2%; *P* < .001). In contrast, follicular lymphoma was less common in inpatients than in outpatients (23% vs 37.1%; *P* < .001). Classical Hodgkin lymphoma made up 18.5% of lymphomas, without differences in its frequency between cohorts. Nodal peripheral T-cell lymphomas were diagnosed in 15.2% of patients, and were more prevalent in inpatients than in outpatients (18.9% vs 13.5%; *P* < .001). Peripheral T-cell lymphoma, NOS, was the main subtype of nodal peripheral T-cell lymphomas (56.3%) and it was more frequent in inpatients than in outpatients (59.4% vs 54.4%; *P* < .001). No differences were observed in the frequency of angioimmunoblastic T-cell lymphoma and anaplastic large cell lymphoma, ALK-negative, between cohorts (Table 2).

### Characteristics of lymphoma subtypes in outpatient and inpatient settings

The characteristics of each subtype of lymphoma in inpatients and outpatients are shown in Tables 3 and 4. In all lymphoma subtypes, inpatients were significantly older than outpatients. Also in all subtypes, lymphadenopathy was less common in inpatients than in outpatients. Although systemic symptoms were more frequent in inpatients than in outpatients, differences did not reach significance in follicular lymphoma. Remarkably, systemic symptoms were the leading clinical manifestation in inpatients with nodal peripheral T-cell lymphoma (46.5%), surpassing lymphadenopathy (38.6%) (Table 4). Furthermore, pain symptoms were more common in inpatients than in outpatients with large B-cell, follicular, and nodal peripheral T-cell lymphomas. In all lymphoma subtypes, the QDU time for diagnosis of outpatients was significantly longer than the admission time for diagnosis of inpatients as it was the time to biopsy of the former compared to the latter (Tables 3 and 4). In addition, inpatients with all subtypes of lymphoma were more likely than outpatients to have a worse performance status, a III-IV Ann Arbor stage, and B symptoms. Whereas an increased LDH was more frequently observed in inpatients than in outpatients with large B-cell, follicular, and nodal peripheral T-cell lymphomas, bulky disease was more common in inpatients than in outpatients with classical Hodgkin and large B-cell lymphomas. Although extranodal disease was more likely in inpatients than in outpatients with all lymphoma subtypes, differences were only statistically significant in large B-cell and nodal peripheral T-cell lymphomas. Inpatients with all subtypes were also more likely than outpatients to have high-risk prognostic scores. Lastly, in all subtypes, there was a minimal rate (≤ 4.1%) of missing data on the variables time to FNAC, time to excisional biopsy, and B symptoms (see footnotes of Tables 3 and 4).

**Table 3:** Characteristics of classical Hodgkin and large B-cell lymphomas in outpatient and inpatient cohorts

	Classical Hodgkin lymphoma (*N* = 318)			Large B-cell lymphoma (*N* = 578)		
Characteristic	OPs *1* (*n* = 216)	IPs *2* (*n* = 102)	*P* value *1* vs *2*	OPs *1* (*n* = 369)	IPs *2* (*n* = 209)	*P* value *1* vs *2*
Age (years), mean (SD)	43.6 (15.8)	51.7 (13.3)	<.001	65.1 (17.5)	69.7 (15.5)	<.001
< 45, *n* (%)	113 (52.3)	44 (43.1)	<.001			
≥ 45, *n* (%)	103 (47.7)	58 (56.9)	<.001			
≤ 60, *n* (%)				121 (32.8)	28 (13.4)	<.001
> 60, *n* (%)				248 (67.2)	181 (86.6)	<.001
Sex, *n* (%)						
Females	98 (45.4)	42 (41.2)	.069	156 (42.3)	84 (40.2)	.104
Males	118 (54.6)	60 (58.8)	.074	213 (57.7)	125 (59.8)	.091
Clinical manifestations, *n* (%)						
Lymphadenopathy	152 (70.4)	51 (50.0)	<.001	246 (66.7)	95 (45.5)	<.001
Systemic symptoms	51 (23.6)	39 (38.2)	<.001	101 (27.4)	86 (41.1)	<.001
Pain symptoms	9 (4.2)	8 (7.8)	.084	11 (3.0)	16 (7.7)	.025
Chest symptoms	3 (1.4)	2 (2.0)	.190	8 (2.2)	8 (3.8)	.120
Other symptoms/signs	1 (0.0)	2 (2.0)	.131	3 (0.8)	4 (1.9)	.135
Successive/first visit ratio	2.10			2.33		
QDU time for diagnosis /admission time for diagnosis (days), mean (SD)	15.5 (1.7)	11.3 (2.7)	.030	16.7 (2.2)	13 (2.5)	.001
Suspected/compatible lymphoma by FNAC, *n* (%)/total *n*	72 (52.9) /136	21 (52.5)/40	.203	156 (71.9)/217	35 (71.4)/49	.155
Time to FNAC (days), mean (SD)	1.3 (0.6)	1.1 (0.4)	.130	1.3 (0.7)	1.2 (0.5)	.125
Time to excisional biopsy (days), mean (SD)	7.3 (1.3)	3.3 (0.9)	<.001	7.1 (1.3)	3.6 (0.9)	.003
ECOG performance score > 1, *n* (%)/total *n*	31 (15.6)/199	21 (21.2)/99	.045	70 (20.8)/336	53 (27.5)/193	<.001
B symptoms, *n* (%)^a^	68 (31.4)	39 (38.2)	.002	107 (29.0)	71 (34.0)	.021
Serum LDH > UNL, *n* (%)	30 (13.9)	18 (17.6)	.082	184 (49.9)	119 (56.9)	<.001
Bulky disease, *n* (%)/total *n*^b^	44 (22.4)/196	27 (28.7)/94	.010	86 (24.9)/345	63 (31.5)/200	.001
Extranodal disease, *n* (%)^c^	33 (15.3)	20 (19.6)	.061			
> 1 extranodal site, *n* (%)				99 (26.8)	69 (33.0)	.009
Ann Arbor stage, *n* (%)						
I-II	142 (65.7)	60 (58.8)	.002	157 (42.5)	71 (34.0)	<.001
III-IV	74 (34.3)	42 (41.2)	.001	212 (57.5)	138 (66.0)	<.001
IPS score (advanced-stage diseased^d^), *n* (%)/total *n*						
Low risk (≤ 3)	166 (84.7)/196	74 (78.7)/94	.035			
High risk (≥ 4)	30 (15.3)/196	20 (21.3)/94	.042			
IPI score, *n* (%)/total *n*						
Low risk (0-1)				114 (33.9)/336	52 (26.9)/193	<.001
Intermediate risk (2-3)				158 (47.0)/336	92 (47.7)/193	.144
High risk (4-5)				64 (19.0)/336	49 (25.4)/193	.004

*Abbreviations*: *OPs* total number of outpatients from the quick diagnosis units of the Hospital Clínic and the Hospital of Bellvitge; *ECOG* Eastern Cooperative Oncology Group; *LDH* lactate dehydrogenase; *UNL* upper normal limit; *IPS* international prognostic score; *IPI* international prognostic index. For other abbreviations, see Table 1

^a^Recurrent fever, night sweats, or > 10% weight loss; ^b^ ≥ 10 cm largest diameter; ^c^Involvement of extra lymphatic tissue; ^d^Advanced-stage disease was defined as stage III or IV disease or stage I or II with bulky disease or stage II disease with B symptoms [59]

For other definitions and explanations, see Table 1 Missing data: variables ‘time to FNAC’ (classical Hodgkin lymphoma: OPs = 5, IPs = 3; large B-cell lymphoma: OPs = 7, IPs = 5), ‘time to excisional biopsy’ (classical Hodgkin lymphoma: OPs = 0, IPs = 1; large B-cell lymphoma: OPs = 3, IPs = 1), ‘B symptoms’ (classical Hodgkin lymphoma: OPs = 2, IPs = 1; large B-cell lymphoma: OPs = 4, IPs = 3)

**Table 4:** Characteristics of follicular and mature nodal peripheral T-cell lymphomas in outpatient and inpatient cohorts

	Follicular lymphoma (*N* = 562)			Nodal peripheral T-cell lymphoma (*N* = 261)		
Characteristic	OPs *1* (*n* = 439)	IP *2* (*n* = 123)	*P* value *1* vs *2*	OPs *1* (*n* = 160)	IP *2* (*n* = 101)	*P* value *1* vs *2*
Age (years), mean (SD)	61.1 (18.4)	65.6 (13.6)	<.001	62.1 (14.3)	68.5 (12.4)	<.001
≤ 60, *n* (%)	212 (48.3)	40 (32.5)	<.001	75 (46.9)	21 (20.8)	<.001
> 60, *n* (%)	227 (51.7)	83 (67.5)	<.001	85 (53.1)	80 (79.2)	<.001
Sex, *n* (%)						
Females	224 (51.0)	59 (48.0)	.071	67 (41.9)	38 (37.6)	.066
Males	215 (49.0)	64 (52.0)	.074	93 (58.1)	63 (62.4)	.068
Clinical manifestations, *n* (%)						
Lymphadenopathy	356 (81.1)	82 (66.7)	<.001	91 (56.9)	39 (38.6)	<.001
Systemic symptoms	52 (11.8)	19 (15.4)	.058	54 (33.8)	47 (46.5)	<.001
Pain symptoms	13 (3.0)	9 (7.3)	.037	10 (6.3)	13 (12.9)	<.001
Chest symptoms	12 (2.7)	8 (6.5)	.054	3 (1.9)	1 (1.0)	.211
Other symptoms/signs	6 (1.4)	5 (4.1)	.086	2 (1.3)	1 (1.0)	.235
Successive/first visit ratio	2.10			2.66		
QDU time for diagnosis /admission time for diagnosis (days), mean (SD)	15.8 (1.8)	11.5 (2.3)	.002	18.3 (2.4)	14.9 (3.0)	.007
Suspected/compatible lymphoma by FNAC, *n* (%)/total *n*	230 (68.5)/336	33 (70.2)/47	.143	45 (58.4)/77	19 (57.6)/33	.215
Time to FNAC (days), mean (SD)	1.4 (0.7)	1.3 (0.5)	.120	1.1 (0.6)	1.2 (0.4)	.184
Time to excisional biopsy (days), mean (SD)	7.6 (1.5)	3.8 (1.0)	.006	7.2 (1.3)	2.9 (0.8)	<.001
ECOG performance score > 1, *n* (%)/total *n*	24 (5.7)/418	12 (10.4)/115	.031	48 (31.6)/152	37 (37.8)/98	.020
B symptoms, *n* (%)	61 (13.9)	24 (19.5)	.020	90 (56.3)	64 (63.4)	<.001
Serum LDH > UNL, *n* (%)	92 (21.0)	34 (27.6)	.003	96 (60.0)	69 (68.3)	<.001
Extranodal disease, *n* (%)	38 (8.7)	14 (11.4)	.093			
> 1 extranodal site, *n* (%)				63 (39.4)	52 (51.5)	<.001
Histologic grading, *n* (%)						
1	123 (28.0)	33 (26.8)	.157			
2	172 (39.2)	48 (39.0)	.208			
3A	114 (26.0)	36 (29.3)	.063			
Unspecified	30 (6.8)	6 (4.9)	.135			
Ann Arbor stage, *n* (%)						
I-II	156 (35.5)	38 (30.9)	.033	36 (22.5)	14 (13.9)	<.001
III-IV	283 (64.5)	85 (69.1)	.034	124 (77.5)	87 (86.1)	<.001
FLIPI score, *n* (%)/total *n*						
Low risk (0-1)	165 (37.6)/439	41 (33.3)/123	.040			
Intermediate risk (2)	153 (34.9)/439	43 (35.0)/123	.214			
High risk (3-5)	121 (27.6)/439	39 (31.7)/123	.049			
IPI score, *n* (%)/total *n*						
Low risk (0-1)				30 (19.7)/152	13 (13.3)/98	.008
Intermediate risk (2-3)				75 (49.3)/152	47 (48.0)/98	.179
High risk (4-5)				47 (30.9)/152	38 (38.8)/98	<.001

*Abbreviations*: *FLIPI* follicular lymphoma international prognostic index

For other abbreviations, definitions, and explanations, see Tables 1 and 3

Missing data: variables ‘time to FNAC’ (follicular lymphoma: OPs = 8, IPs = 5; nodal peripheral T-cell lymphoma: OPs = 4, IPs = 3), ‘time to excisional biopsy’ (follicular lymphoma: OPs = 2, IPs = 2; nodal peripheral T-cell lymphoma: OPs = 1, IPs = 0), ‘B symptoms’ (follicular lymphoma: OPs = 2, IPs = 0; nodal peripheral T-cell lymphoma: OPs = 3, IPs = 2)

### Frequency and characteristics of lymphomas according to referral sources

Potential differences between lymphoma subtypes and their main characteristics between outpatients and inpatients were determined according to the referral sources (Table 5). Outpatients referred from ED were more commonly diagnosed with large B-cell and nodal peripheral T-cell lymphomas than those referred from PC. In contrast, classical Hodgkin and follicular lymphomas were more common among outpatients referred from PC. Moreover*,* outpatients referred from ED were more likely than those referred from PC to have an age > 60 years, a higher frequency of systemic and B symptoms, a worse performance status, and an increased LDH. Also, a III/IV Ann Arbor stage was slightly more common in outpatients referred from ED than from PC, yet without statistically significant differences. Regarding inpatients, those referred from ED were more often diagnosed with large B-cell and nodal peripheral T-cell lymphomas than those referred from PC. In contrast, classical Hodgkin and follicular lymphomas were a more common diagnosis in inpatients referred from PC. Inpatients referred from ED were also more likely than those referred from PC to have an increased LDH. Although the former had a higher frequency of systemic and B symptoms, a worse performance status, and a more advanced Ann Arbor stage than the latter, differences did not reach statistical significance (Table 5).

**Table 5:** Frequency and main characteristics of lymphoma subtypes in outpatient and inpatient cohorts according to referral sources

	OPs (*N* = 1184)			IPs (*N* = 535)		
	PC (*n* = 894)	ED (*n* = 290)	*P* value	PC (*n* = 169)	ED (*n* = 366)	*P* value
Classical Hodgkin lymphoma, *n* (%)	183 (20.5)	33 (11.3)	<.001	41 (24.3)	61 (16.7)	<.001
Large B-cell lymphoma, *n* (%)	242 (27.1)	127 (43.8)	<.001	54 (32.0)	155 (42.3)	<.001
Follicular lymphoma, *n* (%)	370 (41.4)	69 (23.8)	<.001	50 (29.6)	73 (19.9)	<.001
Nodal peripheral T-cell lymphoma, *n* (%)	106 (11.9)	54 (18.6)	<.001	24 (14.2)	77 (21.0)	<.001
Age > 60 years, *n* (%)	451 (50.4)	160 (55.2)	<.001	123 (72.8)	260 (71.0)	.108
ECOG performance score > 1, *n* (%)	120 (13.4)	53 (18.3)	<.001	36 (21.3)	87 (23.8)	.089
B symptoms, *n* (%)	244 (27.3)	93 (32.1)	.001	61 (36.1)	143 (39.1)	.052
Systemic symptoms, *n* (%)	186 (20.8)	72 (24.8)	.012	57 (33.7)	134 (36.6)	.060
Pain symptoms, *n* (%)	33 (3.7)	10 (3.4)	.197	14 (8.3)	32 (8.7)	.210
Serum LDH > UNL, *n* (%)	287 (32.1)	115 (39.7)	<.001	68 (40.2)	172 (47.0)	<.001
Ann Arbor III/IV stage, *n* (%)	520 (58.2)	173 (59.7)	.103	108 (63.9)	244 (66.7)	.066
High risk IPI score (LBCL), *n* (%)	42 (17.4)	22 (17.3)	.230	13 (24.1)	36 (23.2)	.167
High risk IPI score (NPTCL), *n* (%)	32 (30.2)	15 (27.8)	.081	9 (37.5)	29 (37.7)	.231
High risk FLIPI score, *n* (%)	101 (27.3)	20 (29.0)	.098	16 (32.0)	23 (31.5)	.195
High risk IPS score, *n* (%)	25 (13.7)	5 (15.2)	.106	8 (19.5)	12 (19.7)	.252

*Abbreviations*: *PC* primary care; *ED* emergency department; *LBCL* large B-cell lymphoma; *NPTCL* nodal peripheral T-cell lymphoma

For other abbreviations, definitions, and explanations, see Tables 1, 3, and 4

### Results of cost analysis

Table 6 shows the mean costs per day of hospitalization for diagnosis, per outpatient visit, and per patient in inpatients and outpatients. Considering that the mean admission time for diagnosis of inpatients was 12.3 (3.3) days and that the mean number of visits of outpatients (corresponding to the mean QDU time for diagnosis) was 3.26 (1.2), the total cost per hospitalized patient was €4039.56 (513.02), with 69.5% being attributable to personnel salaries and 25.4% to diagnostic tests, and the total cost per outpatient was €1408.48 (197.32), with 50.6% being attributable to diagnostic tests, 29.5% to outpatient visits, and 18.6% to personnel salaries. According to the analysis, the total saving from hospitalization was €2631.08 per patient. There was some degree of missing data on the variables diagnostic tests, therapeutic procedures, pharmaceuticals and consumables, and consultations (see footnote of Table 6).

**Table 6:** Mean costs (€) of outpatients (*n* = 1184) and inpatients (*n* = 535)

Items	Inpatients	Outpatients	Cost per patient (€), mean (SD)		
	One-day stay	One visit	Inpatients^a^	Outpatients^b^	*P* value
Staff salary^c^	228.17^d^	80.52	2806.49 (174.00)	262.50 (22.92)	<.001
QDU (1) and (2) visits^e^	na	127.24	na	414.80 (0.11)	
Diagnostic tests^f^	83.48	218.77	1026.80 (76.00)	713.19 (38.85)	<.001
Therapeutic procedures^g^	1.67	0.54	20.54 (3.53)	1.76 (0.23)	<.001
Pharmaceuticals and consumables	9.95	0.87	122.39 (20.44)	2.84 (0.59)	<.001
Consultations^h^	2.47	0.32	30.38 (9.57)	1.04 (0.37)	<.001
Adverse events	0.50	0.09	6.15 (2.37)	0.29 (0.10)	<.001
Depreciation	2.18	3.70	26.81 (2.44)	12.06 (0.90)	<.001
Total costs	328.42	432.05	4039.56 (513.02)	1408.48 (197.32)	<.001

*Abbreviations*: *na* not applicable. For other abbreviations, see Table 1

^a^Mean (SD) overall admission time for diagnosis: 12.3 (3.3) days

^b^Mean (SD) overall number of outpatients’ visits during the QDU time for diagnosis: 3.26 (1.2)

^c^See Methods for details about salary of staff at inpatient wards

^d^Salary of staff at inpatient wards in charge of 12.5 patients (each ward has 25 beds)

^e^The cost of an average outpatient consultation was based on officially established Catalan Health Service fees

^f^Include costs of laboratory testing and any other investigation (e.g. imaging studies or cytologies and biopsies). The costs of each type of diagnostic tests were based on hospital tariffs in QDU (1) and established fees of the Catalan Health Service in QDU (2)

^g^Include costs of procedures such as therapeutic paracentesis and thoracentesis performed to drain excessive amounts of ascites and pleural fluid, respectively

^h^Include costs of consultations with professionals such as hospital specialists, dieticians, and social workers

Missing data: variables ‘diagnostic tests’ (outpatients = 2.2%, inpatients = 1.5%)¸ ‘therapeutic procedures’ (inpatients = 0.2%)¸ ‘pharmaceuticals and consumables’ (outpatients = 1.7%, inpatients = 1.6%), ‘consultations’ (outpatients = 9.4%, inpatients = 6.8%)

## Discussion

This study revealed that, following referral to hospital, diagnosis of lymphoma is more rapidly accomplished by conventional hospitalization than by hospital-based ambulatory quick diagnostic clinics. Yet the outpatient approach appears to be cost-effective and not detrimental. While no previous study has reported the time to diagnosis and associated costs of an outpatient vs inpatient setting in patients with lymphoma, our study is the first to explicitly describe the clinical and prognostic features of major subtypes of lymphoma according to an outpatient or inpatient diagnosis.

The general features of each lymphoma subtype were consistent with well-known distinctive features. However, there were salient differences between the inpatient and outpatient cohorts. First, lymphoma subtypes in inpatients were more aggressive than in outpatients. Second, inpatients were significantly older and less likely to have lymphadenopathy but more likely in general to have systemic and pain symptoms, worse performance status, advanced Ann Arbor stages, B symptoms, increased LDH, bulky disease, extranodal disease, and high-risk prognostic scores than outpatients. It was of note that the admission time for diagnosis of inpatients was significantly shorter than the QDU time for diagnosis of outpatients. However, because the management of patients referred to outpatient clinics or admitted for investigation of clinical manifestations such as to those reported here and who have an eventual diagnosis of lymphoma can be different in other settings, a circumstance that may depend on various factors such as the type of hospital, the available resources, or the institution traditions, the applicability of the study findings outside Spain is limited, which is an intrinsic limitation. Similarly, although oncologists and hematopathologists - including those from the US - agree that histopathology is essential in lymphoma to make therapeutic decisions [31], excisional biopsy in the US is done much less frequently than in our study (i.e. it is usually performed only after FNAC and only if the diagnosis cannot be reached). Indeed, excisional biopsy is a requirement of the hematology (for QDU (1) outpatients) and the oncology (for QDU (2) outpatients) departments. Therefore, our findings about FNAC/biopsy diagnosis of lymphoma may not be generalizable either.

Although the role of FNAC in the diagnosis of lymphoma is highly controversial [40], several investigators support it as a first, time-saving route for patients with a clinical suspicion of malignancy [41–44], mainly to differentiate metastatic solid cancer from lymphoma lymphadenopathy. In these cases, however, the operator experience in obtaining adequate fine needle aspirates for cytomorphology, flow cytometry, and immunocytochemistry studies is essential [42].

Undeniably, evaluating lymphomas by means of FNAC is challenging. Limitations include sampling error, scarce material to effectively perform flow cytometry and further studies, a relatively high rate of false-negative results, and loss of architecture [9, 40, 42, 45–47]. Nevertheless, it can be a reliable procedure when samples are handled by skilled cytopathologists assisted by specialists in flow cytometry/immunocytochemistry [36, 42].

A former study in 372 consecutive patients referred to QDU (1) for evaluation of peripheral lymphadenopathy revealed malignancy in 120 (32.3%), with an initial fast FNAC being highly specific and sensitive for the diagnosis of metastatic disease. Also, while all patients with lymphoma were fully subtyped by biopsy, a suspicious/compatible diagnosis was initially reached by combining cytomorphology and flow cytometry results in over two-thirds of cases. Owing to insufficient material for flow cytometry or cytomorphology in several cases and the need to perform an excisional biopsy as the unique diagnostic approach, time to diagnosis was significantly longer in lymphoma than in metastatic lymphadenopathy [36]. These findings led us to conclude that a quick FNAC rather than a conventional biopsy can be safely recommended as the initial procedure in patients with suspicious malignant lumps, mostly to differentiate metastasis from lymphomas [36]. In fact, our QDU policy establishes that, if metastatic solid cancer is undisputable by FNAC, an immediate referral to the oncologist will be time-saving, preferably after ordering an imaging examination to locate the primary tumor site if not ostensible before referral. At the first oncologist appointment, they may still order further histological studies for molecular characterization, complete the staging, and decide the best treatment for the patient. Alternatively, if a clearly suspicious/compatible diagnosis of lymphoma is beyond any doubt by FNAC, an immediate referral to the hematologist will also be time-saving, ever ensuring that an excisional biopsy (and ideally a PET-CT scan) has been ordered and scheduled after personal communication with the relevant surgeon. At the first hematologist appointment, the biopsy report will hopefully be ready to read and review and the specialist will likely complete the staging, including a bone marrow biopsy if needed, and decide the best treatment.

Although depending on its subtype, biology and aggressiveness, a delay to diagnosis always constitutes a major concern in lymphomas [48]. The diagnostic interval in secondary care can exceed not only the PC interval but even outdo the treatment interval (i.e. from diagnosis to first treatment) [2]. As a matter of example, a distinct referral pathway for patients referred for lymph node biopsy is frequently lacking in hospital centers [3]. Thus, whereas lymphoma patients most commonly present with peripheral lymphadenopathy - otherwise the strongest predictor of non-Hodgkin and Hodgkin lymphoma in patients aged ≥40 years in PC [1, 10, 48] - service delivery for peripheral lymphadenopathy has been reported to be poorly rationalized [49, 50]. An interesting study conducted in a UK hospital in patients with peripheral lymphadenopathy referred for excisional biopsy revealed that waiting times to it were significantly different according to the referral method [51]. Specifically, patients referred from several sources (mainly the hematology department and general practice) waited a median of 51 days before biopsy when referral was made by post compared to 17 days for faxed referrals and just 4 days when referral was made by direct personal request. A surgical outpatient appointment for routine surgical assessment prior to biopsy was the norm for patients referred by letter, whereas those referred following personal request proceeded straight to biopsy. After this study, and with 43% of biopsies revealing malignancy, the authors’ hospital implemented a fast-track, direct-booking pathway to arrange day-case lymph node biopsies without prior discussion with a surgeon [51].

In our study, 68.4% of inpatients vs 24.5% of outpatients with lymphomas were diagnosed via emergency admission. Because inpatients have preferential access to investigations, PC physicians commonly refer patients with potentially serious conditions directly to the ED in the hope of gaining rapid, including invasive, diagnostic procedures [12, 14]. Disturbingly, some patients referred from PC to the ED are in turn referred from ED to QDU (1) both in this study (data not shown) and in others [11]. Referring patients with suspected malignancy, including lymphoma, to the ED to achieve a quicker access to investigations is not uncommon in countries other than Spain including, among others, the United States (US) [52, 53].

In the UK, during 2006-2013, approximately 27 and 17% of all patients with non-Hodgkin and Hodgkin lymphoma, respectively, were diagnosed after ED presentation [54, 55]. While the survival rates of these patients were significantly lower than those observed in all other routes to diagnosis, the proportion of ED presentations increased with increasing age. Thus, the survival differences were partly explained by the higher proportion of ED presentations among older individuals, as has also been observed in several major types of solid cancer [56]. Although outcomes and survival rates were not investigated in the current study, outpatients and inpatients diagnosed after ED presentation had more aggressive lymphomas, with outpatients referred from ED being older than those referred from PC.

Finally, despite the differences in the time to diagnosis, the remarkable cost differences of the diagnostic evaluation were of note and agreed with findings in studies comparing the costs incurred by QDUs vs admission for disorders such as severe anemia, fever of unknown origin, or lung cancer [12, 13, 25]. A recent cost-minimization study in 63 patients with lymphomas diagnosed at QDU (2) revealed a total cost per hospitalized patient of €5457.42 and of €976.01 per outpatient, meaning a total saving from hospitalization of €4481.41 per patient, or €282,328.83 for the overall sample [25]. Although economic evaluation studies of QDUs are scarce [11–13, 15, 25, 26], savings from hospitalization are overwhelmingly associated with health personnel wages when considering the fractions corresponding to the hours worked.

Strengths of our study included the large sample of patients (*N* = 1779), its duration (10 years), and a design that allowed to analyze representative samples of major lymphoma subtypes according to their aggressive or indolent clinical behavior. Nonetheless, our study should be interpreted in the context of its limitations such as those mentioned above. In addition, and despite the finding of significant savings from hospitalization, there may be multiple factors involved in inpatient admission which were not accounted for in the cost analysis. For instance, aggressive lymphomas are a complex disorder which often require admission for diagnosis due to lymphoma causing severe clinical manifestations other than those described here and that may not be adequately managed in an outpatient unit that is open for 5 h 4 days a week and markedly less staffed. In brief, diagnosis in the hospital may be worth the cost if there are other competing or complementary diagnoses that are managed or treated at the same time. Although detailed clinical, histopathological and cytological information of all inpatients and outpatients were carefully reviewed, certain relevant details might not have been completely captured and potential confounders were not measured - a limitation related to the retrospective design of the study. Furthermore, waiting times to treatment and outcomes were not analyzed, meaning potential differences between cohorts could not be analyzed either. Finally, even though QDUs seem more appropriate for countries with public health care systems, it should be remarked that inpatient admissions in the US - where the health care system is mainly owned by the private sector - also constitute a major component of health costs [57]. A systematic review of Spanish QDUs by US investigators concluded that “[in] our healthcare system with the high cost of inpatient care, the QDU can yield large savings of healthcare dollars while expediting diagnostic workup, increasing patient satisfaction, and preventing lost productivity from hospital stays” [16]. Although some outpatient care is provided by US hospitals in their EDs and specialty clinics, hospitals primarily exist in this country to provide inpatient care. Moreover, ED physicians commonly hospitalize patients for workup [16, 58].

## Conclusion

In summary, among 1779 patients diagnosed with four major subtypes of lymphomas during a 10-year period after referral to hospital-based outpatient diagnostic clinics or hospitalized, clinically aggressive subtypes including large B-cell and nodal peripheral T-cell lymphomas prevailed in admitted patients. For each lymphoma subtype, inpatients were older, had a higher frequency of systemic symptoms than outpatients, and were generally more likely to have a worse performance status, a more advanced Ann Arbor stage, and a higher frequency of B symptoms, increased LDH, bulky disease, extranodal disease, and high-risk prognostic scores. Regardless of the clinical setting, patients referred from EDs had more aggressive lymphoma subtypes than those referred from PCs. Of note, the time to histopathological diagnosis of patients hospitalized for workup was significantly shorter than that of patients evaluated at QDU. Lastly, although there were considerable cost differences between the two settings, there may be other issues associated with admission which may not be properly and safely managed or treated in an outpatient environment and the cost analysis did not account for this potentially added value.

Since outcomes of outpatients and inpatients were not investigated in our study, a challenge of a potential future research is to analyze the impact on outcome of an outpatient vs inpatient diagnostic setting in these patients.

## Abbreviations

ALK: Anaplastic lymphoma kinase
ECOG: Eastern Cooperative Oncology Group
ED: Emergency department
FLIPI: Follicular lymphoma international prognostic index
FNAC: Fine-needle aspiration cytology
ICD-O-3: International Classification of Diseases for Oncology, 3rd edition
IPI: International prognostic index
IPS: International prognostic score
LDH: Lactate dehydrogenase
NICE: National Institute for Health and Care Excellence
NOS: Not otherwise specified
PC: Primary care
PET-CT: Positron emission tomography–computed tomography
QDU (1): Quick diagnosis unit of the Hospital Clínic
QDU (2): Quick diagnosis unit of the Hospital of Bellvitge
QDUs: Quick diagnosis units
SD: Standard deviation
UK: United Kingdom
US: United States
